# Optimizing prediction accuracy in dynamic systems through neural network integration with Kalman and alpha-beta filters

**DOI:** 10.1371/journal.pone.0311734

**Published:** 2024-10-16

**Authors:** Junaid Khan, Umar Zaman, Eunkyu Lee, Awatef Salim Balobaid, R. Y. Aburasain, Muhammad Bilal, Kyungsup Kim

**Affiliations:** 1 Department of Environmental IT Engineering, Chungnam National University, Daejeon, South Korea; 2 Department of Computer Engineering, Chungnam National University, Daejeon, South Korea; 3 Autonomous Ship Research Center, Samsung Heavy Industries, Daejeon, South Korea; 4 School of Computing and Communications, Lancaster University, Lancaster, WA, United Kingdom; 5 Department of Computer Science, College of Engineering and Computer Science, Jazan University, Jazan, Saudi Arabia; Guizhou University of Finance and Economics, CHINA

## Abstract

In the realm of dynamic system analysis, the Kalman filter and the alpha-beta filter are widely recognized for their tracking and prediction capabilities. However, their performance is often limited by static parameters that cannot adapt to changing conditions. Addressing this limitation, this paper introduces innovative neural network-based prediction models that enhance the adaptability and accuracy of these conventional filters. Our approach involves the integration of neural networks within the filtering algorithms, enabling the dynamic augmentation of parameters in response to performance feedback. We present two modified filters: a neural network-based Kalman filter and an alpha-beta filter, each augmented to incorporate neural network-driven parameter tuning. The alpha-beta filter is enhanced with neural network outputs for its *α* and *β* parameters, while the Kalman filter employs a neural network to optimize its internal parameter *R* and noise factor *F*. We assess these advanced models using the root mean square error (RMSE) metric, where our neural network-based alpha-beta filter demonstrates a significant 38.2% improvement in prediction accuracy, and the neural network-based Kalman filter achieves a 53.4% enhancement. Hence, our novel approach of integrating neural networks into filtering algorithms stands out as an effective strategy to significantly enhance their performance in dynamic environments.

## 1 Introduction

The Kalman Filter, introduced by R.E. Kalman in 1960 [1], stands as a cornerstone algorithm for prediction, finding extensive application in fields as varied as GPS positioning, target tracking [2], terrain-referenced navigation (TRN) [3], and spacecraft orbit determination, as well as addressing complex estimation tasks in the Inertial Navigation System (INS) and Global Navigation Satellite System (GNSS) [4]. This algorithm excels at processing time-series data that are often flawed by inaccuracies and noise, enabling the refinement of unknown variable estimations with remarkable precision. Distinguished by its ability for real-time processing, exceptional efficiency, robust resistance to noise, and swift computational estimates, the Kalman Filter has been adopted as a standard for accurate estimation techniques [5]. This has led to its integration into numerous sectors, enhancing technologies in sensor data fusion, integrated navigation, dynamic positioning, and even extending into the realm of microeconomic modeling. In the field of computer vision, the Kalman Filter contributes significantly to edge detection, image segmentation, and pattern recognition, illustrating its versatility and critical role in advancing modern analytical methodologies. However, it is important to acknowledge that despite its numerous advantages, the Kalman Filter algorithm is associated with considerable computational demands [6], which can impact its deployment in resource-constrained scenarios.

The alpha-beta (*α*-*β*) filter algorithm [7–9] is a noteworthy simplification derived from the more complex Kalman Filter, characterized by its minimalistic and straightforward approach. It is essentially the most basic form of an estimator or observer and shares similar functions and areas of application with the Kalman Filter and linear observers. The advantage of the *α*-*β* filter lies in its uncomplicated nature; it bypasses the need to compute the *R* value that is typical in Kalman Filter calculations [10], instead utilizing two primary inputs—alpha and beta—to achieve optimal system accuracy. Furthermore, it requires significantly less storage and computational resources in comparison to the Kalman Filter. Ongoing research is dedicated to accuracy and improving the performance of the Kalman Filter algorithm for a wide array of applications. These enhancements are geared towards improving mathematical robustness and diminishing the computational load [6]. In evaluating the effectiveness of both the Kalman Filter and the alpha-beta filter, there is an intention to employ neural networks as a benchmark to estimate their efficacy against alternative approaches. However, a notable limitation of the Kalman Filter is its intensive computational demand, which may delay its applicability in scenarios requiring real-time processing [10]. Despite this constraint, the algorithm’s proven capability to manage data replete with uncertainty and noise renders it an indispensable instrument in myriad domains of research and industrial innovation.

The alpha-beta (*α*-*β*) filter algorithm [11] is specifically designed for linear systems, performing it ineffective for nonlinear scenarios. Additionally, it depends on the careful tuning of two parameters, alpha and beta, to reach its peak performance. Finding the optimal settings for these parameters can prove to be a daunting and meticulous task, especially in the context of intricate systems. The initial conditions set for the algorithm also play a crucial role in its performance; if they are not accurately determined, the algorithm might not converge to the best estimate. An additional challenge with the alpha-beta filter is its static nature. By integrating a neural network into the alpha-beta filtering system, we can enable dynamic updating of the algorithm’s parameters, shifting from a static approach. This integration facilitates the tuning process, allowing for the parameters to be adjusted more effortlessly.

Neural networks (NN) [12], which draw inspiration from the structure and function of the human brain, have become ubiquitous tools in various domains of machine learning and deep learning. These networks are proficient in tackling tasks that involve either classification or regression. A standard neural network, as described by [13], typically consists of an input layer, several hidden layers, and an output layer. This architecture can be tailored in terms of the number of layers and the number of neurons in each layer to meet the specific needs of the problem at hand.

**Fig 1:**
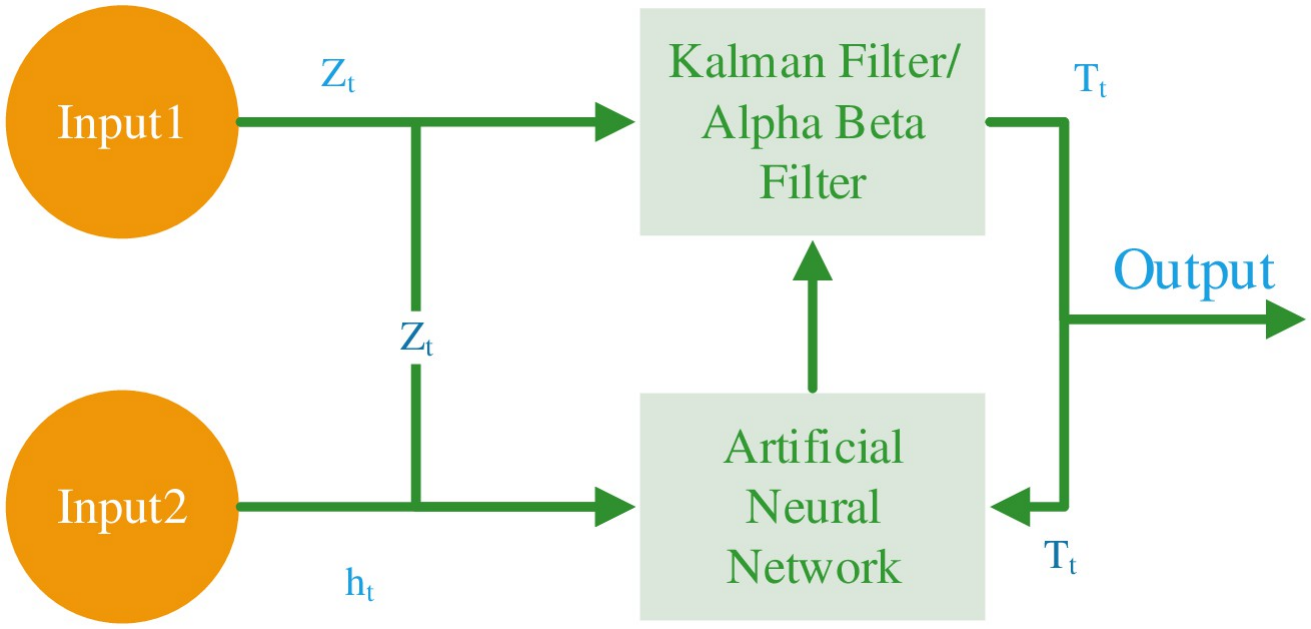
The conceptual model of ANN enabled Kalman filter and alpha beta filter.

In our research, we present advanced and dynamic Kalman Filter and the alpha-beta filter algorithms, both augmented through the integration of neural networks. These enhanced algorithms incorporate neural networks, significantly improving their predictive accuracy and overall functionality. The refined models feature two input layers, specifically designed for processing temperature, humidity, and other sensor data, and are underpinned by a complex structure comprising different hidden layers. The neural network-augmented Kalman Filter incorporates a single output layer, while the modified alpha-beta filter is equipped with two output layers, each accurately structured to optimize the dynamic behavior with the respective filtering algorithm for more accurate predictions. We employ the root mean square error (RMSE) as a performance measure indicator to evaluate the performance of these upgraded algorithms. Our results indicate a noteworthy improvement in both predictive accuracy and operational efficiency, establishing these neural network-enhanced models as superior to the traditional Kalman and alpha-beta filtering methods. In the initial phase of our comparative analysis, we assessed the performance metrics of the Kalman Filter Alpha-Beta Filter. Both filters exhibited high Root Mean Square Error (RMSE) values, with the Kalman and the Alpha-Beta each recording an RMSE of 5.21. The identical initial RMSE values suggest that both filters had a similar degree of inaccuracy, likely resulting from the presence of noise within the input data. Upon integrating neural network learning, both the NN-Kalman and NN-Alpha-Beta filters showed remarkable performance gains. The RMSE of the NN-Kalman filter dropped by 54.22% after fine-tuning specific parameters, showcasing its improved precision in handling complicated data. The NN-Alpha-Beta filter’s RMSE decreased by 38.2%, benefiting from adjustable parameters, thus enhancing its prediction accuracy with a more straightforward approach. Finally, while the NN-Kalman filter excels in detailed and high-accuracy applications, the NN-Alpha-Beta filter stands out for its ease of use and speed, making it suitable for tasks where fast and efficient computation is crucial. Fig 1 shows the conceptual model of ANN enabled Kalman filter and alpha beta filter.

The paper is structured as follows: Section 2 provides a concise overview of relevant literature. Section 3 outlines our detailed methodology, which is divided into three separate subsections. Section 4 presents the results of our study and discusses their implications. Finally, Section 5 concludes the paper.

## 2 Related work

A thorough review of the literature has been conducted, focusing on established techniques relevant to the Kalman filter, the alpha-beta filter, and the broader scope of performance evaluation and prediction. Researchers have recently put forth multiple methods aimed at the advancement and development of filtering techniques, demonstrating relevance to a wide array of practical applications.

Integrating the Kalman Filter (KF) with response control resulted in a filtering process that was both more stable and precise [14]. [15] advanced this development by incorporating an estimated measurement variable into a Gaussian mixture model, using the model’s output as the input for the filter. This fusion, executed via a feedback filter technique, markedly enhanced the accuracy of the filter. [16] presented an approach where the filter’s output, along with the three-state posterior value, was recycled into the system, which effectively mitigated unstable machine oscillations and enhanced the positioning accuracy. A system was introduced where the feedback of the state refined the algorithm by optimizing both the state response advantage and the KF’s input gain, yielding a reduction in tracking errors [17]. A comparative analysis by [18] evaluated parameter estimation through the PI control technique against the standard KF. The findings indicated that both methods delivered similar accuracy, hinting at the potential benefits of their integration in future algorithmic frameworks. [19] discussed a concept known as ‘stacking,’ illustrating how the accuracy of predictive algorithms could be improved through this technique. The evidence pointed out that the accuracy of predictive algorithms was indeed enhanced by employing the stacking method. Finally, [20] developed an innovative ‘mixture-of-experts’ algorithm that adeptly deals with the challenge of combining statistical approximations, which resulted in superior accuracy over methods relying on a single approximation.

[21] presented an adaptive neuro-fuzzy inference technique that leverages gyroscope and acceleration sensors to fine-tune Kalman filter parameters for precise attitude estimation. This method also incorporates a Markov model as an enhancement [22]. [23] introduced an optimal model to improve tracking precision by employing a third-order filter, named due to its use of four parameters: “*α*, *β*, *γ*, and *δ*”. The significance of this filter lies in its incorporation of the ‘jerk,’ the third temporal derivative of the target value, which also allows for the prediction of the variable’s second-order derivative. The authors argue that this inclusion of both second and third-order derivatives greatly enhances the precision of the tracking filter. In a separate advancement, the author in [8] developed the DELM to enhance the *α*-*β* filter algorithm’s precision. Collectively, these research findings illustrate that Kalman and alpha-beta filter algorithms are robust in managing uncertain and noisy datasets. Additionally, enhancements and the creation of hybrid algorithms can further refine their performance. The integration of neural networks with these filters can also escalate their accuracy and effectiveness.

An effective prediction tool must be capable of recognizing and adapting to changes in its environment. This adaptability can be achieved through a learning component that allows the tool to continually improve its performance. Past research has shed light on similar advancements. For example, [24] improved the Kalman filter’s accuracy for determining a robot’s position by integrating a fuzzy system, a method that proved especially useful in dynamic conditions where sensor noise is prevalent. They addressed the increased noise by adding extra sensors that tracked the robot’s movement, thus enhancing the fuzzy system’s ability to refine the Kalman filter’s output.

[7–9, 25] has integrated an alpha-beta filter with various learning methodologies, including Deep Extreme Learning Machine (DELM), Support Vector Machine (SVM), Deep Belief Network (DBN), and Feed-Forward Artificial Neural Network (FF-ANN), to evaluate performance enhancements within the alpha-beta filter. Each method yielded distinct results. In the comparison of these algorithms, DELM proved to be the most complex. Notably, they achieved considerable performance improvements with the FF-ANN method. However, comparing this model to other methods, such as the Kalman filter, remains a complex task. Over time, the Kalman filter has seen several improvements [26–28]. A more sophisticated iteration has been developed to address complex, non-linear problems, providing more accurate estimates and reducing errors [29]. The ensemble Kalman filter has been introduced to manage situations with many variables [30–32], while a fusion approach combines the Kalman filter with other techniques to further diminish errors [33].

The paper presents a novel prediction model that boasts advanced learning capabilities, adapted for flexibility across changing conditions. Unlike traditional models with fixed frameworks, our pioneering solution is capable of managing several trained models concurrently. It demonstrates the adaptability to transition between these models in response to different environmental factors, maintaining consistent accuracy. Optimized for particular scenarios, each model can be engaged or interchanged according to environmental cues, ensuring reliable prediction accuracy even as conditions fluctuate.

## 3 Methodology

In this section, we explain the methodology behind our proposed system. Traditionally, each algorithm undergoes training using historical data, during which it discovers hidden patterns and the correlations between the data points to forecast results. However, these prediction algorithms typically do not have the capacity to learn from static data; they are geared towards dynamic data instead. To address this limitation, we have proposed a Kalman filter [34] and alpha beta filter algorithms [9] that are grounded in neural network technology.

**Fig 2:**
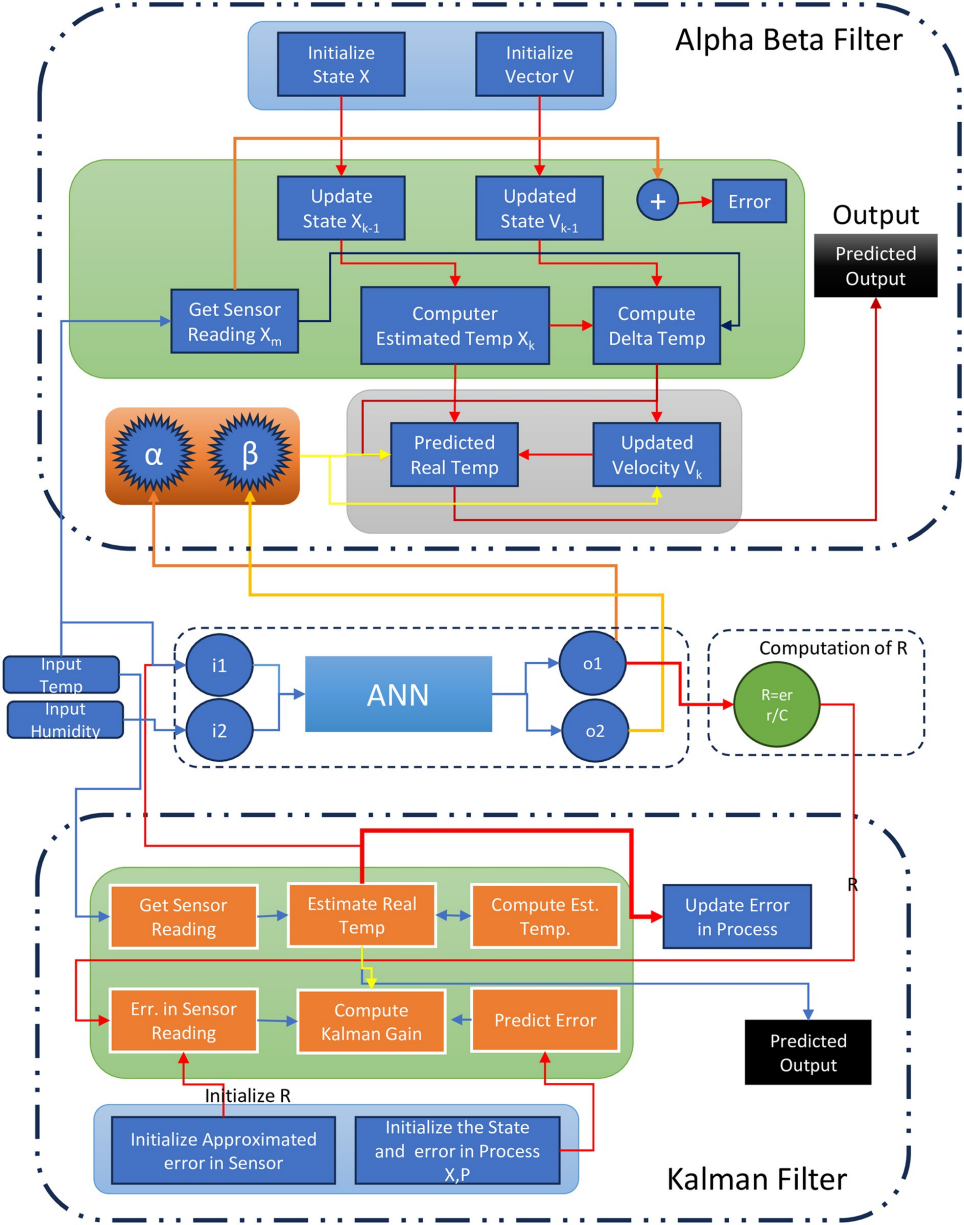
A comprehensive proposed learning to prediction model for neural network based kalman filter and alpha beta filter.

The upper portion of Fig 2 illustrates a neural network-based alpha-beta filter, wherein the input and hidden layers are identical. The output layer produces two parameters, alpha and beta, which are then inputted into the alpha-beta filter to predict the final result. A significant limitation of this algorithm is that the values of alpha and beta are static. To address this, we are attempting to introduce dynamism to the filter by incorporating a neural network.

The lower portion of Fig 2 illustrates the neural network based Kalman filter, which features two input values and a varying configuration of hidden layers, terminating in an output layer tasked with calculating the R-value. This approach, which we refer to as the neural network learning method, involves receiving inputs—namely, the previously predicted temperature, the current temperature, and the current humidity. These inputs are processed by the Artificial Neural Network (ANN) [35–37] before being fed into the Kalman Filter (KF) algorithm. The output from the NN represents the predicted error in sensor readings. To estimate the error in these readings, we divide it by a constant factor, denoted as (F), which is the R-value. This R-value is then fine-tuned by the Kalman Filter algorithm in order to adjust the Kalman gain (KG) [38] appropriately, thereby enhancing the accuracy of its predictions. Our proposed model enables the Kalman filter to accurately deduce the actual temperature from noisy sensor data by dynamically adjusting to the error rate. Fig 2 depicts the proposed model that combines the neural network with the Kalman and alpha-beta filters.

In our experiment, we used the Kalman filter to make predictions, along with a learning module supported by artificial neural networks. The Kalman filter is known for being a straightforward yet effective tool, needing only the previous state’s data, not a complete history, to make smart guesses about the system’s current condition. We used this filter to get a clearer reading of temperatures, reducing the noise that can interfere with temperature sensors. This noise often comes from situations where the readings are significantly impacted by the moisture in the air.

We used an artificial neural network (ANN) for the learning module, designed to handle three inputs: the current temperature, the expected temperature (feedback), and the level of moisture in the air. The Kalman filter works in real time and looks at the temperature sensor’s reading at a specific moment, marked as *z*_*t*_, to give a cleaned-up temperature, *T*_*t*_, by filtering out the noise. The performance of the Kalman filter is adjusted by changing the Kalman gain (K), a setting that is updated in every step, based on the process covariance matrix (P) and the estimated error in the sensor readings (R).

The main function of the learning module is to fig out the error in the sensor readings (R) to smartly adjust the Kalman gain (K). In the following subsection, we will provide a concise overview of the Kalman filter’s operation and its pivotal role in this experiment.

### 3.1 Kalman Filter algorithm

**Fig 3:**
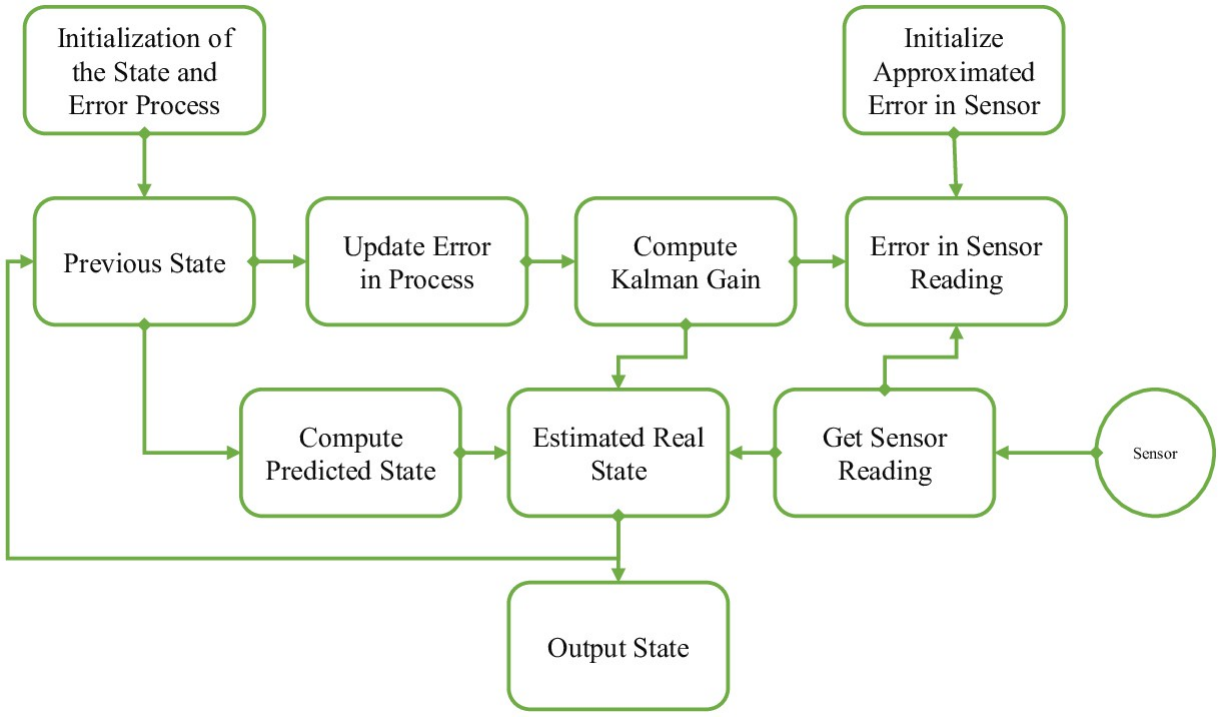
The complete structure of Kalman filter algorithm.

The Kalman Filter is renowned for its efficiency and capability to perform optimally without requiring extensive historical data. It utilizes information from the most recent known state to formulate intelligent predictions about the system’s present state. A critical component of this model is the Kalman Gain (K), which serves to reconcile the system’s internal forecasts with incoming data from sensors, thus ensuring a balanced approach. The complete structure of Kalman filter algorithm is shown in Fig 3.

Noise from the environment is a common problem that can make sensor readings less accurate. In this study, we’re looking at a temperature sensor, denoted as *T*_*t*_, that’s affected by this kind of noise. The Kalman filter uses a special model to make an educated guess about the system’s state (like an estimated temperature). It then compares this guess with the actual data from the sensor to come up with a more accurate temperature reading for the next moment in time *T*_*t*+1_.

An initial step involves the calculation of the temperature predicted, *T*_*P*_, derived from the predecessor value estimated through Eq 1:

$$\begin{array}{c} T_{P}=X\cdot T_{t-1}+Y\cdot u_{t} \end{array}$$ (1)

Here, *T*_*P*_ represents the predicted temperature internally, while *X* and *Y* are the state transition and control matrices, respectively. *T*_*t*−1_ is the prior temperature reading, and *u*_*t*_ is the control vector.

Uncertainty in this internal prediction is quantified by a covariance factor, updated as Eq 2:

$$\begin{array}{c} P_{\text{predicted}}=X\cdot P_{t-1}\cdot X^{T}+Q \end{array}$$ (2)

Where *X* and *X*^*T*^ are the state transition matrix and its transpose, *P*_*t*−1_ is the preceding covariance value, and *Q* denotes the estimated process error.

Subsequent to this, the Kalman gain, *K*, undergoes an update via Eq 3:

$$\begin{array}{c} K=\frac{P_{\text{predicted}}\cdot H^{T}}{H\cdot P_{\text{predicted}}\cdot H^{T}+R} \end{array}$$ (3)

Here, the observation matrix and its transpose signify by *H* and *H*^*T*^, with *R* denoting the estimated error measurement.

Assuming the sensor’s reading temperature at time *t* is *z*_*t*_, the refined estimated temperature is obtained through Eq 4:

$$\begin{array}{c} T_{t}=T_{\text{Predicted}}+K\left(z_{t}-H\cdot T_{\text{Predicted}}\right) \end{array}$$ (4)

Conclusively, the covariance is updated for subsequent iterations via Eq 5:

$$\begin{array}{c} P_{t}=\left(1-K\cdot H\right)P_{\text{predicted}} \end{array}$$ (5)

This explanation highlights how well the Kalman Filter can reduce noise, ensuring accurate measurements. This is especially important in settings where sensor readings can be easily distorted or affected by surrounding conditions. The complete algorithm is also presented in Algorithm 1.

#### 3.1.1 Neural network for Kalman Filter

Consider a neural network model that includes *H* input neurons and a hidden layer with *N* neurons. The value of the *n*-th neuron in the hidden layer is computed as the weighted sum of the input neurons plus a bias term. In a three-layer Artificial Neural Network (ANN), the computations for the hidden and output layer neurons are formalized by the subsequent Eqs 6–8:

The input to the *n*-th neuron in the hidden layer is given by:

$$\begin{array}{c} v_{n}=\sum_{i=1}^{H}a_{ni}\cdot x_{i}+b_{n} \end{array}$$ (6)

where *v*_*n*_ is the input to the *n*-th neuron in the hidden layer, *a*_*ni*_ signifies the weight from the *i*-th neuron in the input layer to the *n*-th neuron in the hidden layer, *x*_*i*_ represents the *i*-th input variable, and *b*_*n*_ denotes the bias for the *n*-th neuron in the hidden layer. The outputs of the hidden layer neurons are then transformed using an activation function.

The output of the *n*-th neuron after applying the activation function is:

$$\begin{array}{c} h_{n}=f\left(v_{n}\right) \end{array}$$ (7)

where *h*_*n*_ designates the output of the *n*-th neuron in the hidden layer, and *f* is the activation function, such as Sigmoid, Tanh, ReLU, etc.

The output for the *k*-th neuron in the output layer is computed as:

$$\begin{array}{c} y_{k}=\sum_{n=1}^{N}c_{kn}\cdot h_{n}+d_{k} \end{array}$$ (8)

where *y*_*k*_ denotes the output of the *k*-th neuron in the output layer, *c*_*kn*_ is the weight from the *n*-th neuron in the hidden layer to the *k*-th neuron in the output layer, and *d*_*k*_ is the bias associated with the *k*-th output neuron.

**Algorithm 1** ANN Enabled Kalman Filter

1: **procedure** PredictTemperature(*z*_*t*_, *h*_*t*_)   ⊳ Inputs: temperature and humidity readings at time *t*

2:  **Start**

3:  *z*_*t*_ ← Temp Sensor   ⊳ Collect temperature reading

4:  *h*_*t*_ ← Humidity Sensor   ⊳ Collect humidity reading

5:  **Process temperature reading using Kalman Filter:**

6:   Use *z*_*t*_ as input to the Kalman Filter Algorithm

7:   Output predicted temperature *T*_*t*_ and error estimate *R*

8:  **Process readings using Artificial Neural Network (ANN):**

9:   **Input:** *z*_*t*_ (temperature reading), *h*_*t*_ (humidity reading), and *R*

10:   **Output:** refined temperature prediction (*T*_*t*_)

11:  **return** Predicted Temperature (*T*_*t*_)

12:  **End**

13: **end procedure**

### 3.2 Integrated alpha beta filter

The *α*-*β* filter is renowned for its simplicity and efficiency and is widely used in various fields for estimation, control, and smoothing purposes. It serves as a less complex yet effective alternative to advanced filtering methods such as the Kalman filter. The *α*-*β* filter’s strength lies in its ability to operate without requiring a detailed system model, enabling faster calculations and requiring less memory space, while still offering improved performance over traditional linear filters.

In the modern adaptation of the *α*-*β* filter, a neural network is integrated to optimize the values of *α* and *β* dynamically. This neural network takes temperature and humidity as inputs, enhancing the filter’s adaptability and precision in real-time temperature prediction scenarios. The neural network is trained to output optimized *α* (NN_*α*_) and *β* (NN_*β*_) values, ensuring that the filter’s responsiveness is tuned to the specific characteristics influenced by the prevailing environmental conditions.

The application of the integrated *α*-*β* filter with a neural network is predicated on the assumption that a system’s dynamics, influenced by variables like temperature and humidity, can be sufficiently represented by a two-state model at separate intervals. The neural network-enabled alpha beta filter setup is given in algorithm 2.

The filter’s implementation involves several steps, beginning with initialization, as outlined below.

The neural network takes the current temperature *T* and humidity *H* as inputs and outputs optimized values for *α* and *β*. We denote the outputs of the neural network as NN_*α*_ and NN_*β*_ as represented in Eqs 9 and 10:

$$\begin{array}{c} {\text{NN}}_{\alpha }=f\left(T,H\right) \end{array}$$ (9)

$$\begin{array}{c} {\text{NN}}_{\beta }=g\left(T,H\right) \end{array}$$ (10)

Here, *f* and *g* represent the neural network’s processing functions that determine *α* and *β*, respectively, based on the given temperature and humidity.

With the dynamically optimized *α* and *β* values, the *α*-*β* filter’s equations for predicting temperature are as follows: First, we need to do initialization. We set the initial estimated temperature and rate of change of temperature, as shown in Eqs 11 and 12:

$$\begin{array}{c} T_{0}=\frac{c_{1}}{c_{2}} \end{array}$$ (11)

$$\begin{array}{c} r_{0}=\frac{c_{1}}{c_{2}} \end{array}$$ (12)

For Temperature Update and Data Incorporation, we update the temperature and incorporate sensor data, illustrated in Eqs 13 and 14:

$$\begin{array}{c} T_{1}=T_{0}+r_{0}\times \Delta t \end{array}$$ (13)

$$\begin{array}{c} T_{m}=T_{1} \end{array}$$ (14)

Here, Δ*t* represents the time interval, and *T*_*m*_ is the measured temperature.

For Temperature Difference and Prediction, we calculate the difference in temperature and update the temperature estimate using the dynamically optimized *α* from the neural network, shown in Eqs 15 and 16:

$$\begin{array}{c} d_{T}=T_{m}-T_{1} \end{array}$$ (15)

$$\begin{array}{c} T_{1}=T_{1}+{\text{NN}}_{\alpha }\times d_{T} \end{array}$$ (16)

For the Rate of Change of Temperature Prediction and Update, We predict and update the rate of change of temperature using the dynamically optimized *β* from the neural network, as described in Eqs 17–19:

$$\begin{array}{c} r_{1}=r_{0}+{\text{NN}}_{\beta }\times \frac{d_{r}}{\Delta t} \end{array}$$ (17)

$$\begin{array}{c} T_{2}=T_{1} \end{array}$$ (18)

$$\begin{array}{c} r_{2}=r_{1} \end{array}$$ (19)

**Algorithm 2** Neural Network Enabled Alpha Beta (*α*-*β* Filter)

1: **Input:** Current temperature *T*, humidity *H*

2: **Output:** Optimized values NN_*α*_ and NN_*β*_

3: Compute the optimized *α* and *β* using the neural network:

4:  NN_*α*_ = *f*(*T*, *H*)

5:  NN_*β*_ = *g*(*T*, *H*)

6: Initialize the estimated temperature and rate of change of temperature:

7:  $T_{0}=\frac{c_{1}}{c_{2}}$

8:  $r_{0}=\frac{c_{1}}{c_{2}}$

9: Update the temperature and incorporate sensor data:

10:  *T*_1_ = *T*_0_ + *r*_0_ × Δ*t*

11:  *T*_*m*_ = *T*_1_

12: Compute the difference in temperature and update the temperature estimate:

13:  *d*_*T*_ = *T*_*m*_ − *T*_1_

14:  *T*_1_ = *T*_1_ + NN_*α*_ × *d*_*T*_

15: Predict and update the rate of change of temperature:

16:  $r_{1}=r_{0}+{\text{NN}}_{\beta }\times \frac{d_{r}}{\Delta t}$

17:  *T*_2_ = *T*_1_

18:  *r*_2_ = *r*_1_

### 3.3 Effectiveness of Kalman and alpha-beta filters in dynamic system

Kalman and *α*-*β* filters are highly effective in handling dynamic system changes due to their inherent adaptability in processing time-series data with uncertainty and noise. The Kalman filter, in particular, is designed for systems where real-time data is constantly updated, as it utilizes a recursive approach to make predictions based on previous states and current measurements. This is critical in dynamic environments like temperature monitoring, where factors such as humidity and external noise can distort sensor readings.

The *α*-*β* filter, while simpler, operates efficiently in real-time systems by smoothing noisy sensor data and quickly responding to sudden changes. These characteristics make both filters suitable for temperature prediction applications, where the system must adapt to rapid environmental changes. In contrast to traditional machine learning models, which require vast amounts of historical data and are often unable to adjust in real time, these filters excel in dynamic, noisy environments. They are particularly effective in this study because temperature and humidity are naturally variable and unpredictable over time.

By combining these filters with neural networks, the dynamic response of the filters is enhanced. The neural network compensates for the static nature of the *α*-*β* filter by dynamically adjusting the *α* and *β* parameters based on real-time data, making it responsive to changes in environmental conditions. In the case of the Kalman filter, the neural network helps fine-tune the error estimates, thereby improving the overall prediction accuracy in fluctuating temperature conditions. This dynamic adaptation is crucial for minimizing data bias and improving the filter’s ability to respond to changing system dynamics, especially in the field of temperature prediction.

## 4 Experimental results and discussion

For our study, we utilized actual temperature and humidity data gathered over a year in Seoul, South Korea. The genuine readings of temperature and humidity, sampled every hour from January 1 to December 31, 2010. The dataset used in this paper was taken from [8]. The dataset consisted of a total of

365×24=8760

records. To assess the relationship between the real temperature and humidity metrics, we used the Pearson correlation coefficient [39, 40] as given in Eq 20:

$$\begin{array}{c} \text{Rel}\left(X,Y\right)=r=\frac{\sum \left(x_{j}-\bar{x}\right)\left(y_{j}-\bar{y}\right)}{\sqrt{\sum {\left(x_{j}-\bar{x}\right)}^{2}\sum {\left(y_{j}-\bar{y}\right)}^{2}}} \end{array}$$ (20)

Where Rel(*X*, *Y*) represents the correlation coefficient *r* between temperature (*X*) and humidity (*Y*). The symbols *x*_*j*_ and *y*_*j*_ stand for the temperature and humidity for the jth hour. The average temperature and humidity are symbolized by $\bar{x}$ and $\bar{y}$ respectively.

We noticed a small positive link between humidity (shown as *Y*) and temperature (shown as *X*). This was confirmed by *r*(8758) = 0.22 and *p* < 0.0001. To make the data more varied, we changed the temperature readings based on the humidity data. This change was done randomly, but it was tied to the current average humidity, as shown in Eq 21:

$$\begin{array}{c} \left|Err\right|\propto \frac{y_{j}-\bar{y}}{y_{max}-\bar{y}} \end{array}$$ (21)

Here, |*Err*| is the change in the temperature readings. *y*_*j*_ is the current humidity, *y*_*max*_ is the highest humidity, and $\bar{y}$ is the average humidity.

Using this change, the temperature readings became as shown in Eq 22:

$$\begin{array}{c} T_{\text{simple}}=\frac{y_{j}-\bar{y}}{y_{\text{max}}-\bar{y}}\times R\left(-1,1\right)\times S+X_{\text{real}} \end{array}$$ (22)

Here, *T*_*simple*_ is the changed temperature reading. R picks a random value between -1 and +1. *S* changes the size of the error, and *X*_*real*_ is the actual temperature. Table 1 shows the Summary of the collected data.

**Table 1:** Summary of collected and simulated noisy data.

	Min	Max	Avg	Stdev
Temperature (°C)	-15.30	33.40	12.14	11.39
Sensor Readings (°C)	-21.36	38.58	12.16	12.58
Humidity (g/m^3^)	12.00	97.00	62.90	19.89
Sensor Error	0.00	10.00	5.99	2.34

**Fig 4:**
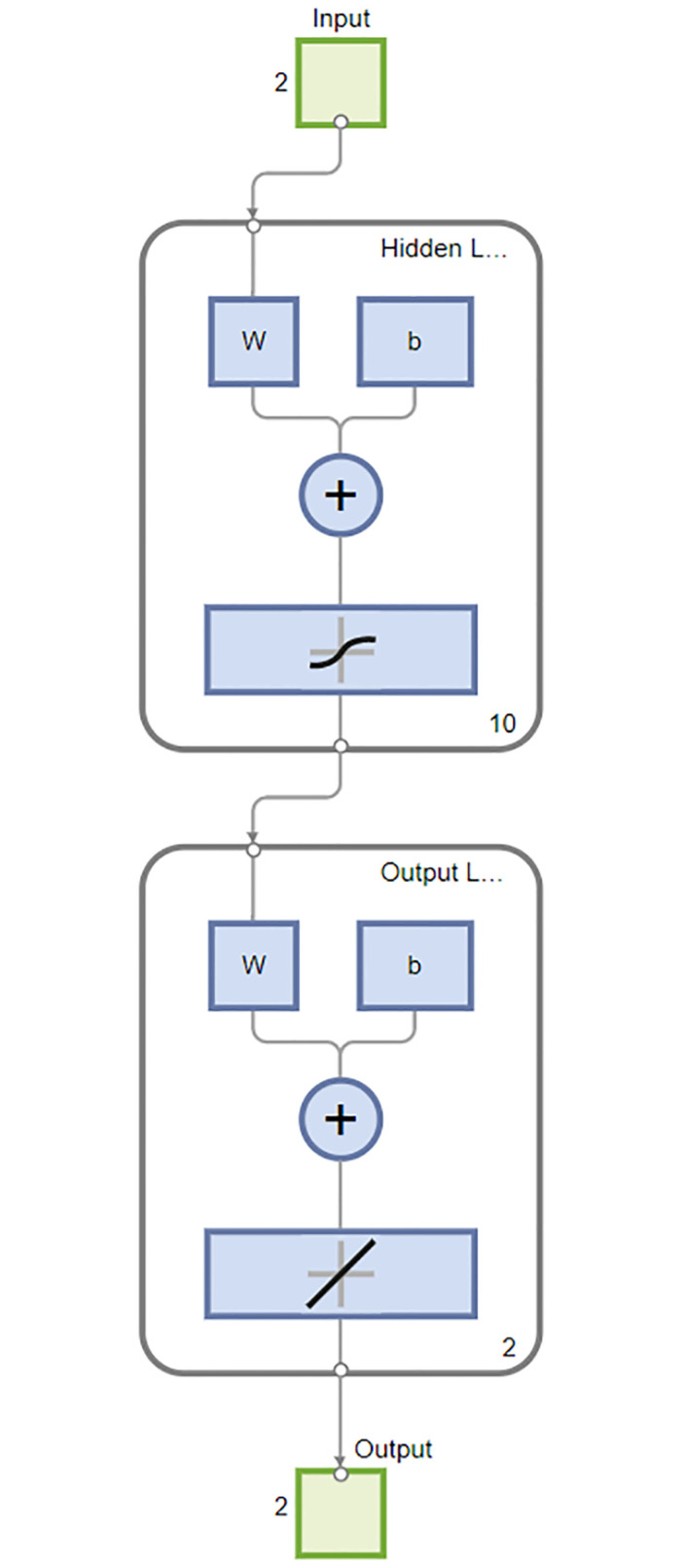
The experimental setup for the neural network includes two input layers designated for the alpha-beta filter and the Kalman filter.

The Fig 4 depicts the experimental configuration of the neural network, which comprises two input layers for the alpha-beta filter and three input layers for the Kalman filter. Each algorithm incorporates a hidden layer with 15 neurons. The alpha-beta filter is equipped with two output layers, whereas the Kalman filter employs a single output layer dedicated to R computation. Subsequent to the data processing via the neural network, the outputs were calibrated to fine-tune the performance of both the Kalman filter and the alpha-beta filter for additional calculations.

### 4.1 Cross validation

Before inputting data into the Kalman filter and alpha-beta filter, we utilized different configurations for the Artificial Neural Network (ANN). The ANN parameters were then passed to the respective filters. The training of the ANN algorithm involved exploring various configurations by altering the number of neurons in the hidden layer, the activation function, and the learning rates. To ensure robust evaluation and mitigate the stochastic nature of ANN weight initialization, multiple independent experiments were conducted for each configuration. The results reported are averages, thus reducing the influence of randomness.

**Fig 5:**
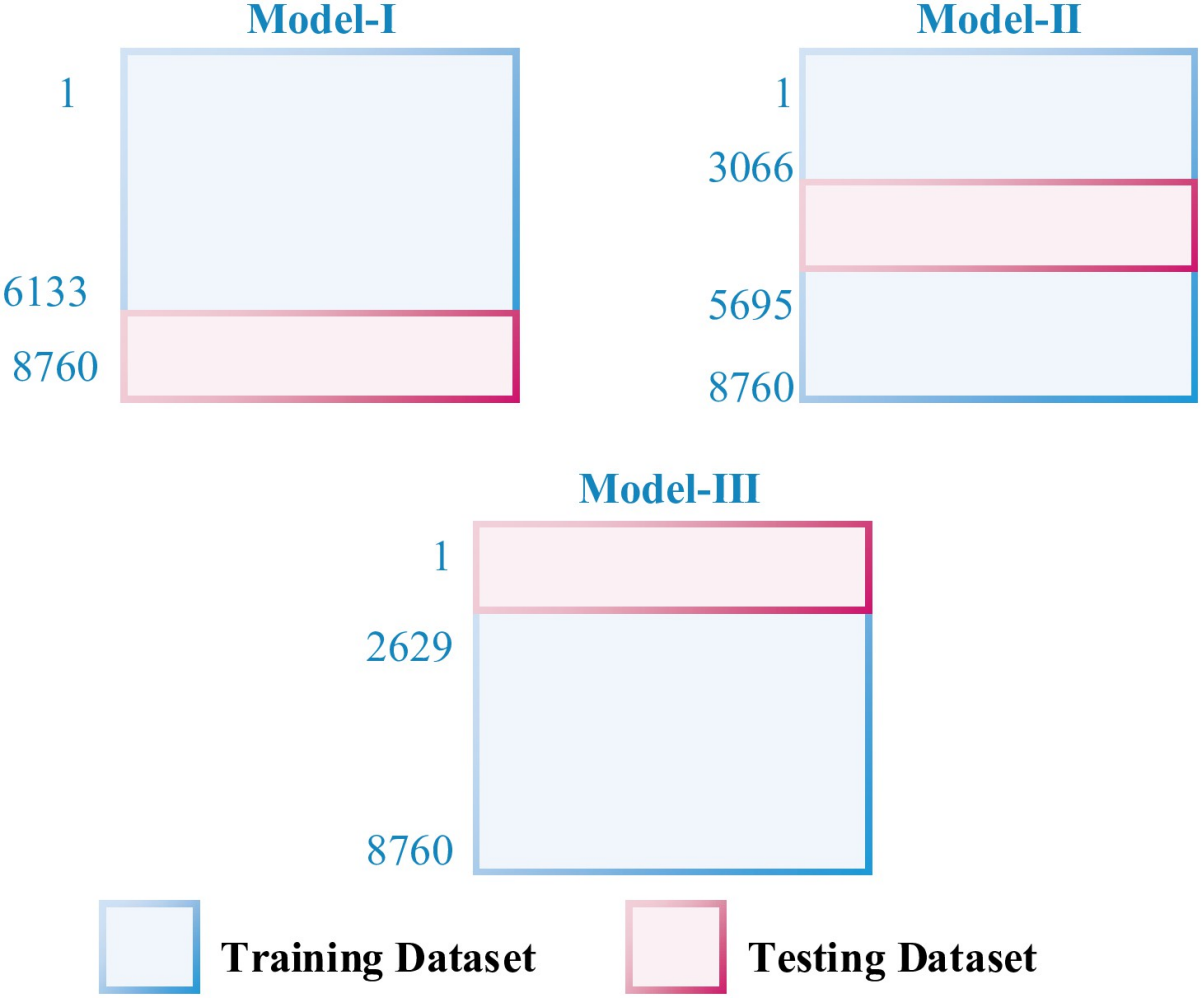
3 fold cross validation model for the ANN training and testing data.

To prevent training bias, we employed a 3-fold cross-validation technique. This approach involved dividing the dataset into four subsets of equal size. As depicted in Fig 5, 75% of the data was allocated for training, while the remaining 25% was used for testing the ANN algorithm across different configurations. The training process utilized the Levenberg–Marquardt algorithm, renowned for its efficiency and speed in training moderately-sized neural networks. The maximum number of epochs for training was set at 300.

The results indicate that using a linear activation function led to minimal impact on the ANN’s performance concerning changes in the number of neurons in the hidden layer or the learning rate. However, significant variations in prediction accuracy were observed across different models during the 3-fold cross-validation process.

### 4.2 Performance evaluation metrics

The Root Mean Squared Error (RMSE) [41–43] serves as a vital metric in statistical modeling and machine learning, particularly when gauging the accuracy of prediction models. It offers an aggregate measure of the magnitude of error between predicted and observed values. Mathematically defined, given predictions ${\hat{y}}_{i}$ and actual values *y*_*i*_ across *i* = 1, 2, …, *n* observations, the RMSE is articulated in Eq 23:

$$\begin{array}{c} \text{RMSE}=\sqrt{\frac{1}{n}\sum_{i=1}^{n}{\left(y_{i}-{\hat{y}}_{i}\right)}^{2}} \end{array}$$ (23)

This formulation is intrinsically tied to the L2 norm or the Euclidean distance in the space of error magnitudes. By squaring the individual errors, the RMSE inherently places higher weight on larger discrepancies, emphasizing the detrimental effects of outliers. The subsequent square root transformation ensures the error remains on a scale comparable to the original data, fostering more intuitive interpretations.

One might naturally compare the RMSE to the Mean Squared Error (MSE), its unscaled counterpart as shown in Eq 24:

$$\begin{array}{c} \text{MSE}=\frac{1}{n}\sum_{i=1}^{n}{\left(y_{i}-{\hat{y}}_{i}\right)}^{2} \end{array}$$ (24)

While the MSE [44] captures variance in errors, its squared units can sometimes obfuscate real-world interpretations. The RMSE alleviates this concern, rendering the error back to the data’s original units, and thus providing a more direct sense of model accuracy.

In many empirical settings, the RMSE stands out as a preferred metric because of its sensitivity to large errors, its direct interpretability, and its grounding in the geometric principles of the L2 norm.

### 4.3 Implementation of Kalman filter

**Fig 6:**
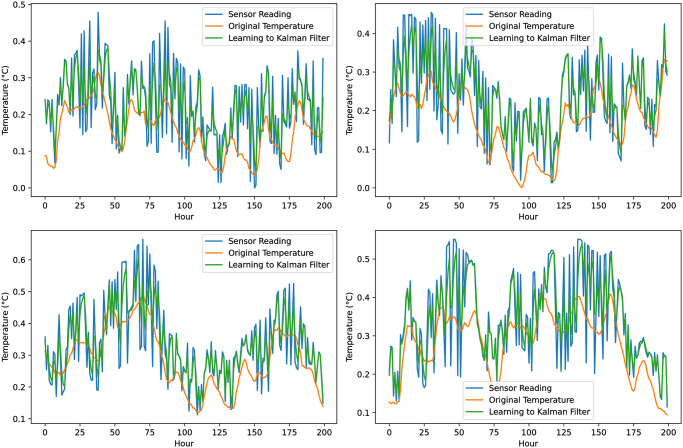
The temperature sensor readings with the original temperature and learning to Kalman filter algorithm prediction results (each plot represent results for 200 data instances).

We developed and implemented the proposed system to assess the Kalman filter algorithm’s performance with a learning module in Python. The system was tested using a dataset encompassing a year’s worth of temperature and humidity readings, along with simulated noisy sensor data. This dataset, sourced from an external text file, was integrated into the application’s internal database. The dataset incorporated four variables: the actual temperature, the noisy sensor reading, humidity, and error value. Fig 6 shows the temperature sensor readings with the original temperature and learning to Kalman filter algorithm prediction results.

Our initial step involved calculating the root mean squared error (RMSE) of the sensor readings by comparing them with the real temperature values. The initial sensor reading values in terms of RMSE were very high such as 5.216.

Our experiment was conducted with different adjustable *R* values and collected the results to check and compare the accuracy with each *R* value. However, with the Kalman filter application, the accuracy improved compared to traditional methods, reducing the error to 2.43 while setting the varying *R* value and *F* value of 0.005, where *F* is the error factor, we were able to achieve a notable improvement in our predictive accuracy, as evidenced by a 53.41% reduction in the root mean square error (RMSE). However, this result, while promising, suggests that further optimization still needs improvement.

**Fig 7:**
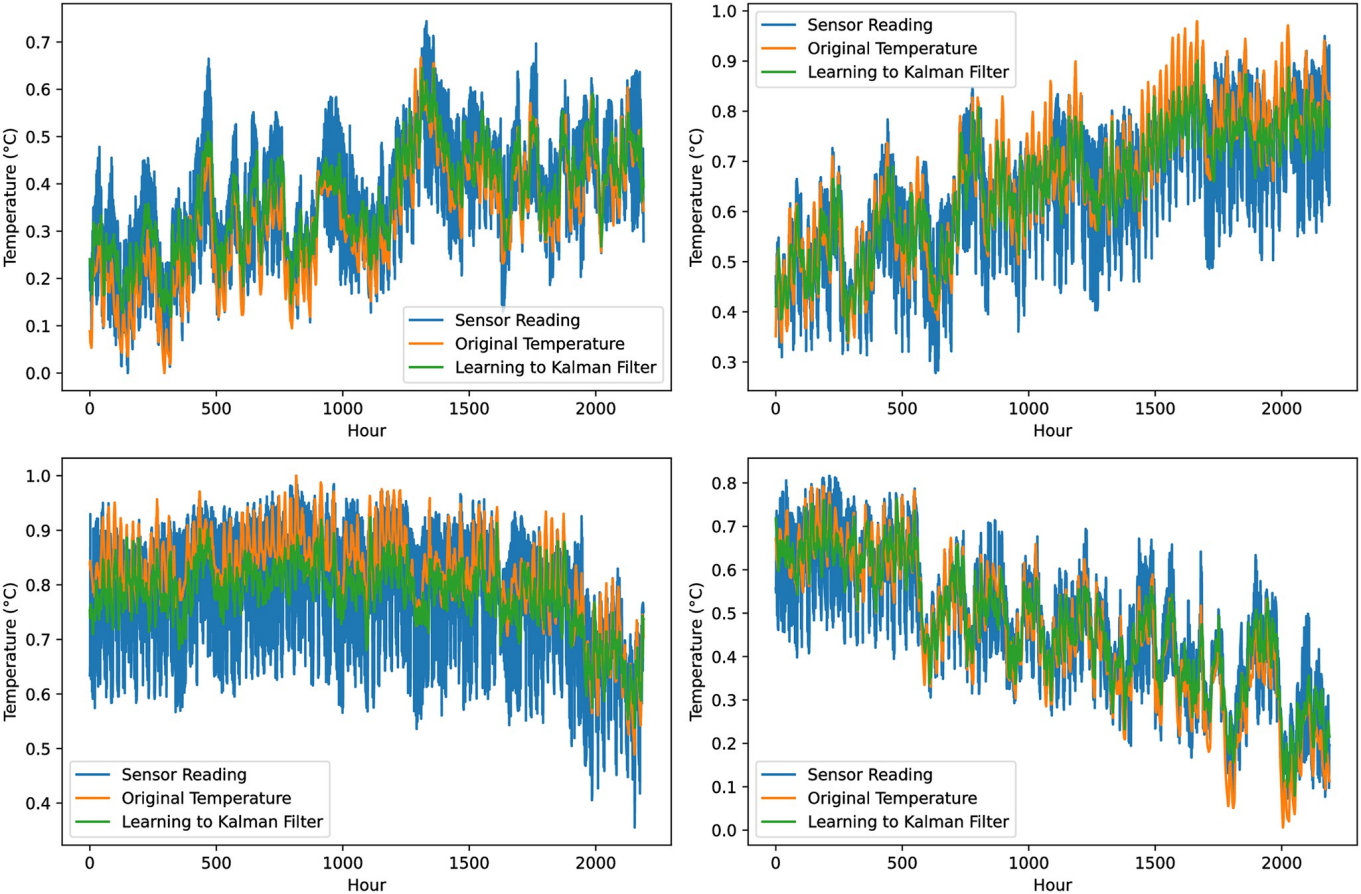
Details prediction results for learning to Kalman filter, each subfigure results for 2000 data instances.

Fig 7 details prediction results for learning to Kalman filter, each subfigure results for 2000 data instances. Adjustments to *R* and the noise factor *F* were made to analyze results under varied conditions. The most favorable result was attained with *R* set to 20 and *F* value to 0.02, achieving an error reduction to 2.388 from 5.216, another promising result was achieved with a notable improvement in our predictive accuracy, as evidenced by a 54.22% reduction in the root mean square error (RMSE). To address improvement in Kalman filter, we made use of the *Filterpy* library in Python to implement an Artificial Neural Network (ANN) learning-based approach. This ANN was constructed to consider variables such as humidity data, predicted temperature estimates, and sensing data, with the primary aim of refining our prediction of potential sensor errors. It is of note that, to ensure the integrity and consistency of our ANN input data, normalization techniques were applied. Table 2 shows the Kalman filter prediction results with and without the ANN-based learning module in terms of RMSE and MSE.

**Table 2:** Kalman filter prediction results with and without the ANN-based learning module in terms of RMSE and MSE.

		Kalman filter with ANN					
Metric	Sensing Data	F = 0.005	F = 0.008	F = 0.01	F = 0.02	F = 0.05	F = 0.1
RMSE	5.216	2.432	2.410	2.402	2.388	2.417	2.481
MSE	27.204	5.914	5.807	5.770	5.701	5.844	6.157

The enhancement percentage was determined using the Eq (20):

$$\begin{array}{c} \text{Percentage}\;\text{Decrease}=\frac{\left|\text{Starting}\;\text{Value}-\text{Final}\;\text{Value}\right|}{\text{Starting}\;\text{Value}}\times 100 \end{array}$$ (25)

This ANN was constructed to consider variables such as humidity data, preliminary temperature estimates, and sensing data, with the primary aim of refining our prediction of potential sensor errors. It is of note that, to ensure the integrity and consistency of our ANN input data, normalization techniques were applied as given in Eq 26. This neural model had two input neurons for humidity, sensor readings, and forecasted temperature, along with one output neuron for error prediction in sensor readings.

$$\begin{array}{c} x_{j}=\frac{x_{j}-x_{\text{low}}}{x_{\text{high}}-x_{\text{low}}} \end{array}$$ (26)

Here, *x*_*j*_ is the standardized value of the jth data point related to the variables of input and output. *x*_low_ and *x*_high_ represent the minimum and maximum values, respectively, for each parameter.

Due to the data normalization in the neural system, it’s essential to revert the network’s output using Eq 27:

$$\begin{array}{c} e_{j}=e_{j}^{*}\times \left(e_{\text{high}}-e_{\text{low}}\right)+e_{\text{low}} \end{array}$$ (27)

For the neural system’s training, various configurations were explored, including changes in the neuron count within the hidden layer, activation functions, and learning rates. In accordance with this method, 75% of data was designated for training, and the remaining 25% for validation. The neural training process, with a maximum epoch count set at 100.

The presented results show that utilizing a linear activation function in the Artificial Neural Network (ANN) results in marginal sensitivity to changes in the neuron count of the hidden layer and variations in the learning rate. Nevertheless, distinct disparities in predictive accuracy were evident across different training and validation models. Significantly, the model with *R* = 20 and *F* = 0.02 demonstrated superior accuracy. While the sigmoid activation function is a popular selection in ANN designs, our data accentuates a consistent improvement in predictive accuracy over its linear counterpart. The optimal results were observed and were attained using the ANN with a sigmoid activation function, 10 neurons in the hidden layer, and a learning rate of 0.2. This specific configuration was subsequently adapted to enhance the performance of the Kalman filter algorithm.

The predicted error rate, when compared with the inherent data error, underscores the rigorous training of our learning module on the supplied dataset. As highlighted earlier, *R* denotes the anticipated measurement error and is directly correlated with the expected error rate in sensor readings. In mathematical terms:

$$\begin{array}{c} R=\frac{err_{i}}{F} \end{array}$$ (28)

In this expression, *F* represents the proportionality constant, commonly referred to as the error factor.

**Fig 8:**
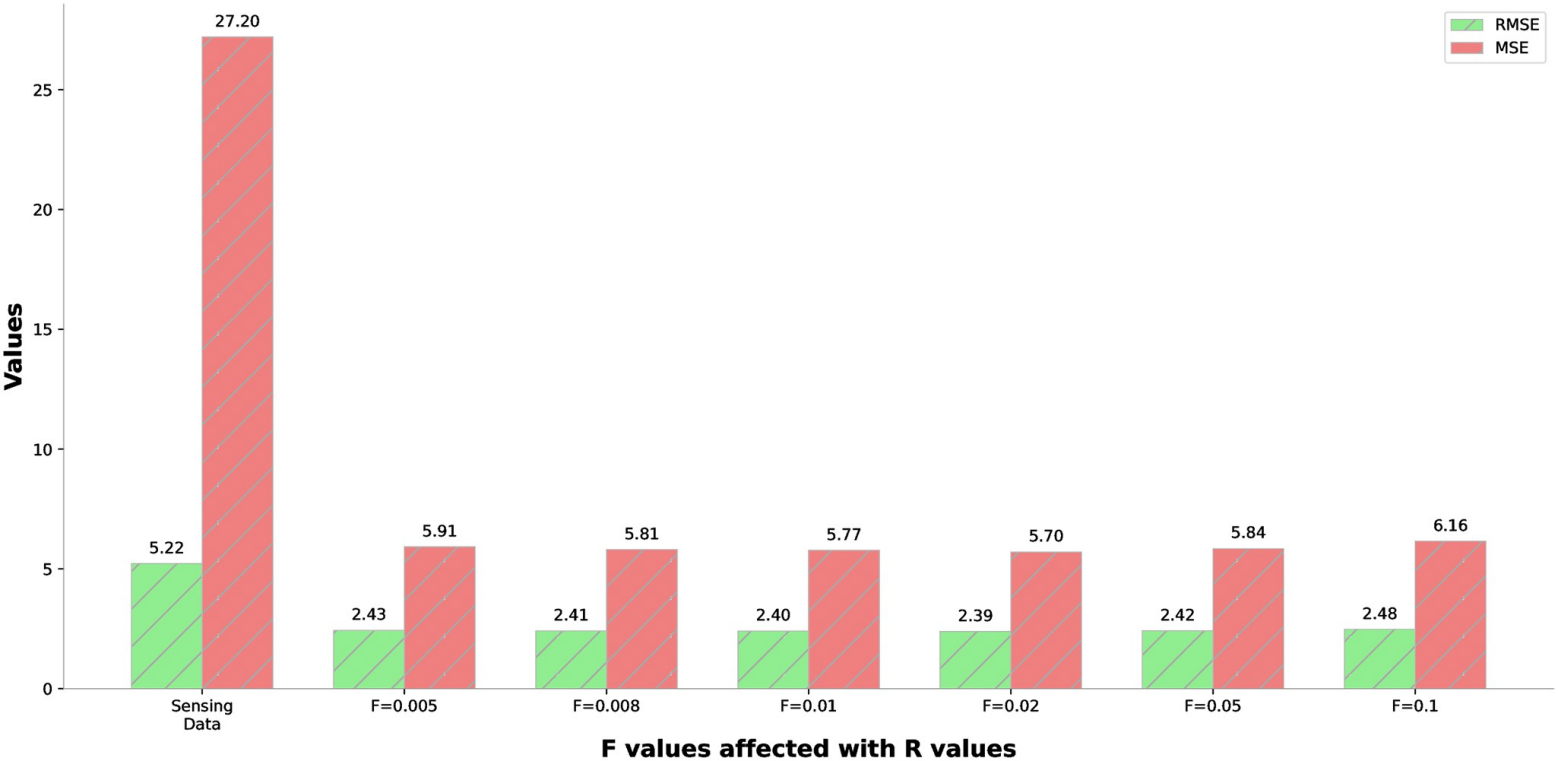
Kalman filter prediction results with and without the ANN-based learning module in terms of RMSE and MSE.

In our performance evaluation, we compared the predictive results of the traditional Kalman filter algorithm with those of our proposed predictive learning model, aiming to determine the enhancements in the Kalman filter’s predictive accuracy. For the standard Kalman filter, results were determined across varied values of *R*. The optimal setting for *R* remained non-static, depending upon the specific dataset we have. Determining the most suitable value for *R* in the Kalman filter manually poses a significant challenge. Consequently, we assumed experiments spanning a range of *R* values and noted variations in the Kalman filter’s predictive accuracy correlating with these variations. Fig 8 shows the Kalman filter prediction results with and without the ANN-based learning module in terms of RMSE and MSE

**Fig 9:**
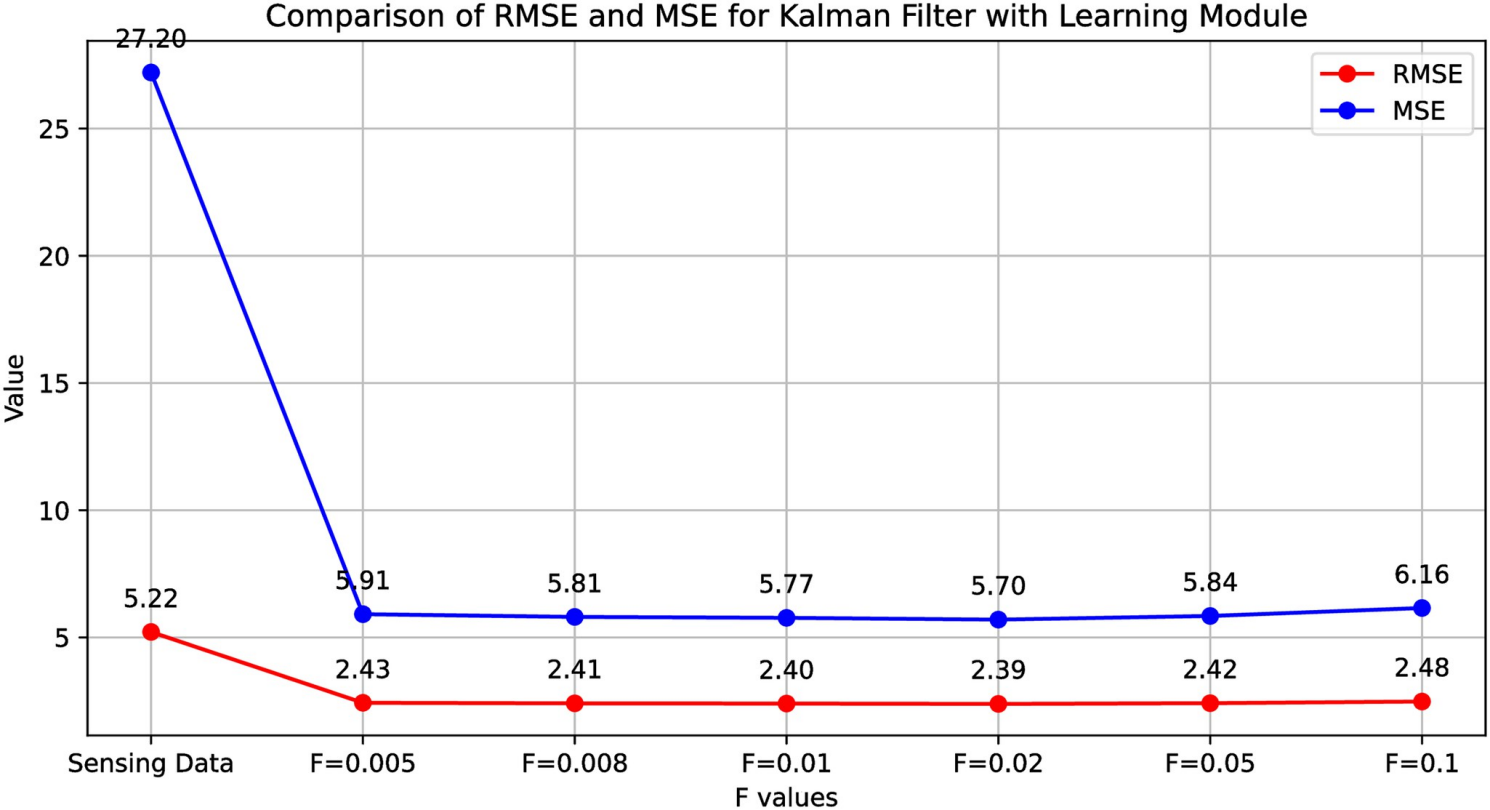
Comparison of the error reduction in terms of RMSE and MSE for Kalman filter with learning module.

We now discuss the results of the Kalman filter adjusted using our learning model. After we trained the ANN module, we used it to make the Kalman filter work better by adjusting its *R* and *F* parameters. As we mentioned earlier, to determine *R* from the expected error, we have to pick a suitable value for *F*. This *F* value is the proportionality constant, also called the error factor, which we referred to in Eq 28. So, we did some tests by changing the values of *F* to see what works best. So, to make it easier to compare, we used some simple statistical measures to turn these results into single numbers. Fig 9 shows the comparison of the error reduction in terms of RMSE and MSE for the Kalaman filter with learning module.

### 4.4 Implementation of alpha beta filter

In this research, a system with two primary components—a learning module and a prediction module—was developed. The implementation was performed on a computer equipped with an 11th Gen Intel Core i7-11700KF processor with a 3.60GHz CPU and 32GB of RAM, utilizing Python 3.12.0. The dataset contained hourly records of temperature and humidity, alongside simulated noisy sensor data over the span of a year. A notable root mean square error (RMSE) of 5.21 in the noisy sensor data indicated significant inaccuracies.

The study revealed that altering the number of neurons in the hidden layers of artificial neural networks (ANNs) had minimal influence on performance. The most accurate results were attained with an ANN featuring ten neurons in the hidden layer, using a sigmoid activation function, and integrating an alpha-beta filter.

To evaluate the sensor data’s reliability, a comparison was made between the actual temperature values and the noisy readings, summarized in Table 1 of the paper. An alpha-beta filter was employed to address the substantial observed discrepancies, resulting in an improved RMSE of 3.21.

Subsequently, a learning unit was incorporated into the alpha-beta filter to boost its efficacy. This enhancement was facilitated by employing an ANN to dynamically modify the alpha-beta parameters based on historical data. The data underwent preprocessing for normalization prior to its introduction into the ANN, followed by post-processing for de-normalization. The equations utilized for this process are depicted as follows:

In data processing, normalization is a crucial step for scaling values within a specified range to ensure uniformity in the behavior of data-driven algorithms. To normalize data points, we utilize the following equation:

$$\begin{array}{c} Scaled\left(x\right)=\frac{value(x)-minimum(x)}{maximum(x)-minimum(x)} \end{array}$$ (29)

Here, *Scaled*(*x*) represents the normalized value for the *x*th data point, *value*(*x*) denotes the current value to be normalized, and *minimum*(*x*) and *maximum*(*x*) correspond to the minimum and maximum observed values in the dataset for that specific point, respectively.

For processes requiring the retrieval of original data values from normalized figures, a de-normalization procedure is applied, which is defined by the equation:

$$\begin{array}{c} Original(x)=Scaled(x)\times (maximum(x)-minimum(x))+minimum(x) \end{array}$$ (30)

Eq 29 systematically transforms each value to a common scale, while Eq 30 facilitates the conversion of these normalized figures back to their original scale, ensuring that the transformation is reversible and data integrity is maintained throughout the process.

**Fig 10:**
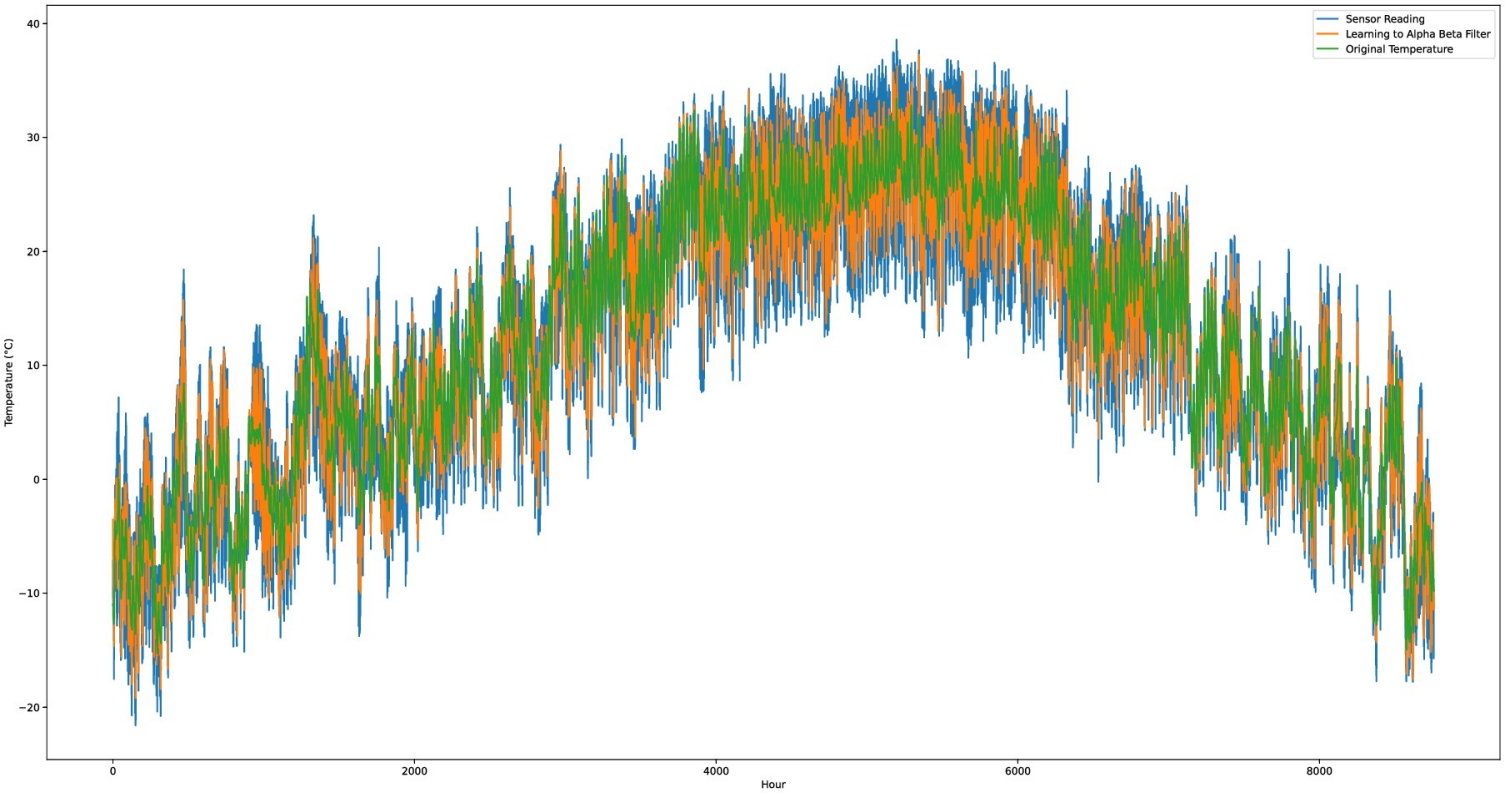
Learning to alpha beta filter prediction results with original temperature data and sensor readings.

Fig 10 illustrates the discrepancies among the sensor readings, the temperature values predicted by the learning-enhanced alpha-beta filter, and the actual temperature values. The predictions made by the learning-enhanced alpha-beta filter were significantly closer to the real temperature values than those derived from the traditional alpha-beta filter.

The input layer of our neural network receives two data points: humidity and temperature. We experimented with different neuron counts in the hidden layer, specifically 5, 10, 15, 25, and 30 neurons, to assess their impact on the model’s performance. The configuration that yielded the highest accuracy utilized 15 neurons in the hidden layer. The output layer produces two values, alpha and beta, which are then processed through an alpha-beta filter to generate the forecasted results. In this model, we apply sigmoid activation functions in the hidden layer and a linear activation function in the output layer. This type of neural network is referred to as a multilayer perceptron, capable of approximating any measurable function with the desired level of accuracy.

The RMSE and MAE for the original sensor readings using the traditional alpha-beta filter and the learning-enhanced alpha-beta filter are depicted in Fig 9. Introducing a learning facet to the alpha-beta filter yielded a remarkable 38.45% enhancement in prediction accuracy, as gauged by the RMSE metric. The derived statistical measures unequivocally affirm that the proposed learning-enhanced alpha-beta filter substantially outstrips the conventional alpha-beta filter in terms of performance.

Table 3 compares two different configurations of the *α*-*β* filter with a learning algorithm and without a learning algorithm. The performance of these configurations is evaluated using two different metrics: RMSE (Root Mean Square Error) and MAE (Mean Absolute Error).

**Table 3:** Performance comparison of *α*-*β* filters with and without ANN.

	With ANN	Without ANN	Percentage Increase
**RMSE**	3.22	5.21	38.2%
**MAE**	2.53	3.95	35.9%

#### 4.4.1 *α*-*β* filter with learning algorithm

The RMSE value is 3.22, which represents the square root of the average squared differences between the predicted and actual values. A lower RMSE value indicates a more accurate model, and in this context, an RMSE of 3.22 suggests that when the learning algorithm is applied, the filter’s predictions are fairly close to the actual data points on average.

The MAE value is 2.53, which measures the average magnitude of the errors in a set of predictions, without considering their direction (positive or negative errors are treated equally). A lower MAE value signifies that the filter’s predictions, on average, deviate less from the actual values, pointing to its strong predictive accuracy.

#### 4.4.2 *α*-*β* filter without learning algorithm

The RMSE is considerably higher at 5.21, indicating less accurate predictions compared to the learning-integrated counterpart. This higher value signifies that the predictions are, on average, more spread out from the actual values, leading to larger squared differences.

**Fig 11:**
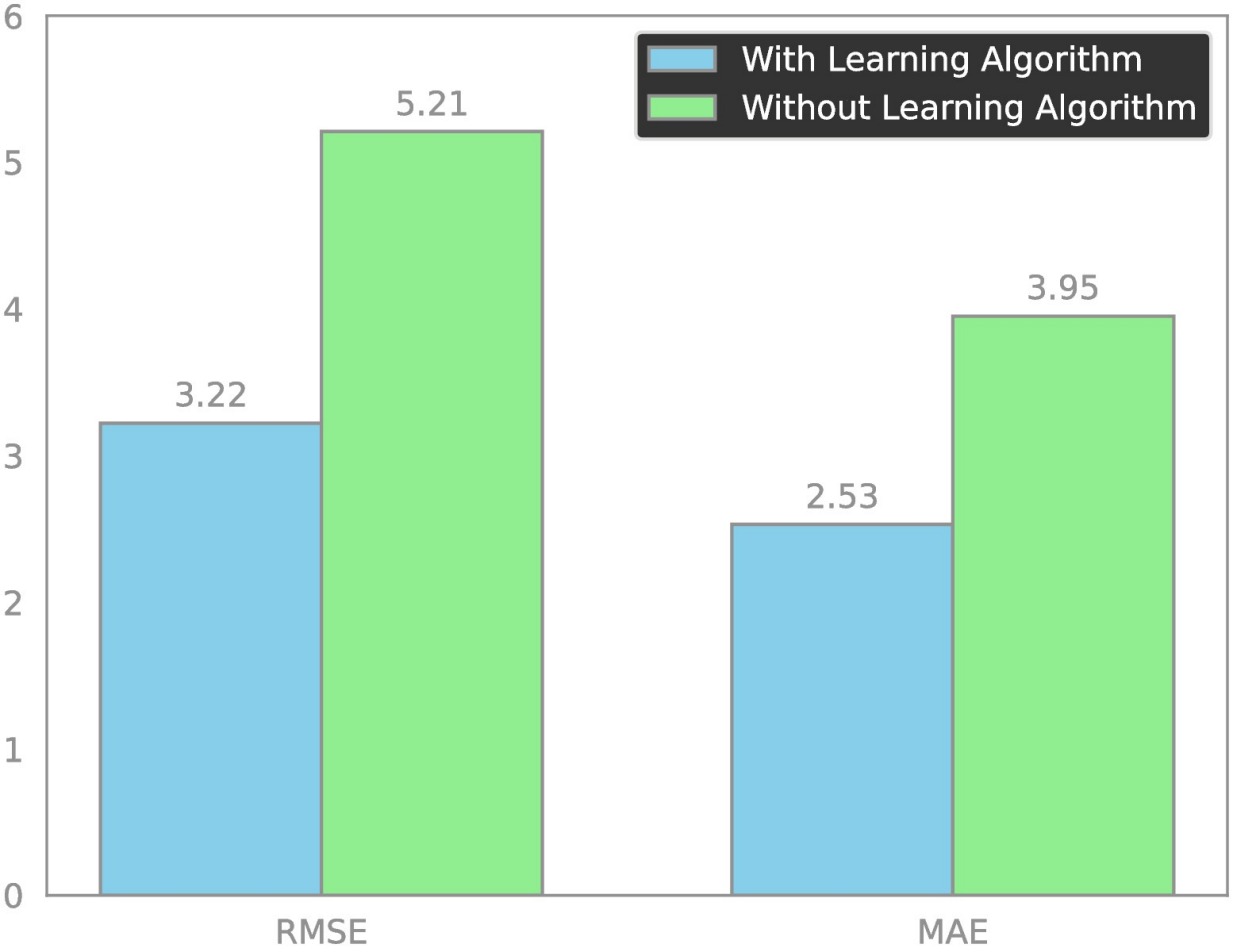
Performance comparison of *α*-*β* filters with and without ANN.

The MAE is also higher at 3.95, which further confirms that the predictions are less precise and have greater absolute errors on average. The performance comparison of *α*-*β* filter algorithm with and without ANN is shown in Fig 11.

#### 4.4.3 Percentage increase in performance

The RMSE improvement is quantified as a 38.2% increase in performance, which is a substantial uplift. This percentage reflects how much more accurately the *α*-*β* filter with the learning algorithm predicts the target variables compared to the non-learning version.

The MAE sees a similar improvement at 35.9%, indicating a significant enhancement in the predictive accuracy by reducing the average error magnitude.

The implications of these numbers are crucial for understanding the impact of incorporating learning algorithms into filtering methods. The *α*-*β* filter, a predictive tool commonly used for tracking and estimating variables over time, relies on two parameters (*α* and *β*) to adjust its predictions. The integration of a learning algorithm allows the filter to adaptively modify these parameters based on the incoming data, enabling it to correct its predictions over time as more data becomes available.

The table’s figs explicitly demonstrate that the adaptive learning version of the *α*-*β* filter is superior to the static version. This superiority is evidenced not just by a marginal enhancement but by substantial percentages, highlighting the effectiveness of the learning algorithm in reducing prediction errors.

**Fig 12:**
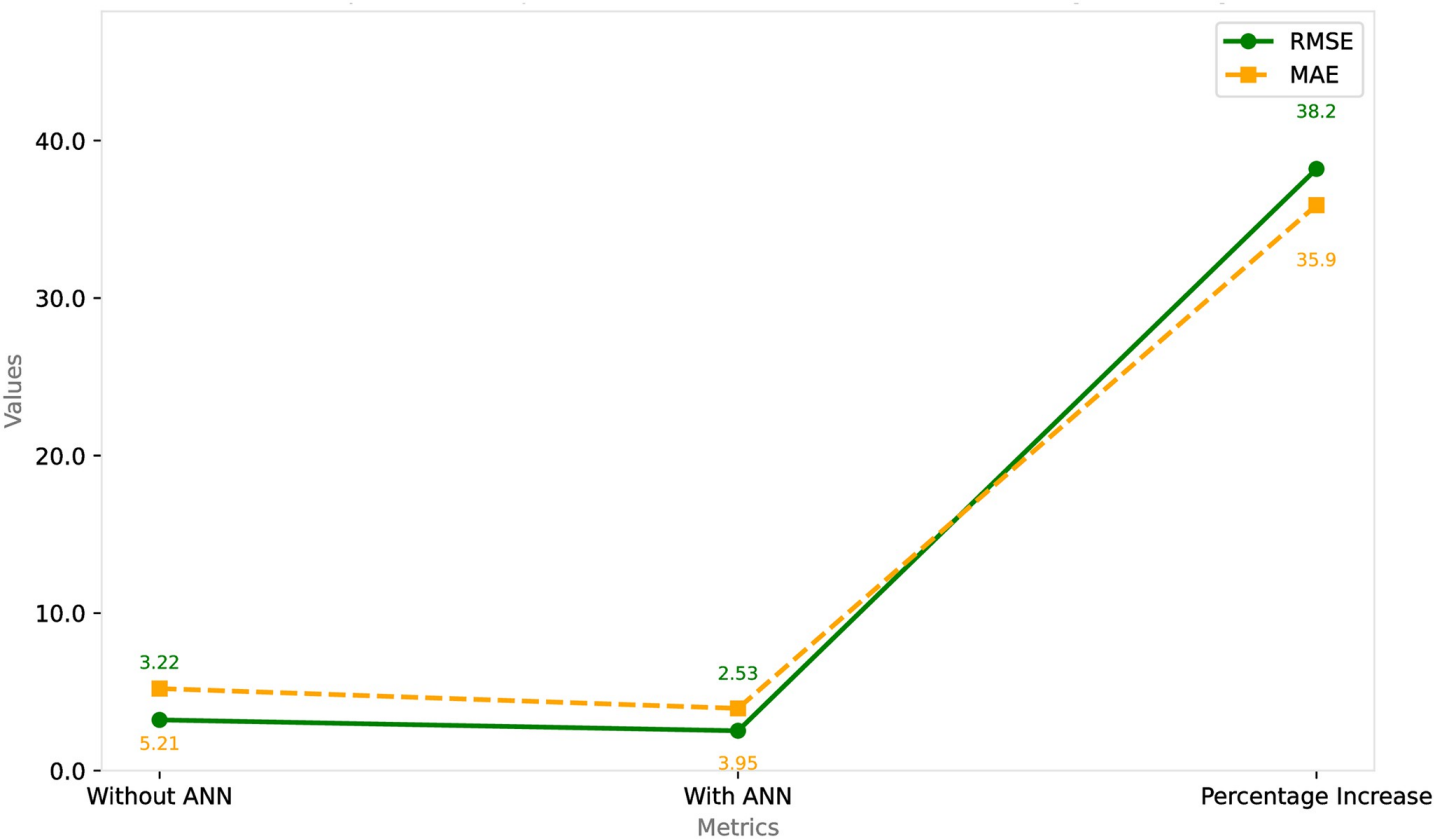
Performance comparison of *α*-*β* filter with and without ANN, including percentage increase.

This performance increase is critical in fields that demand high precision, such as navigation, tracking, and time series forecasting. The improvements seen in the RMSE and MAE indicate that the model is not only making fewer large errors (as seen by the RMSE reduction) but also fewer errors on average (as seen by the MAE reduction), leading to more trustworthy predictions. It’s these kinds of advances that can make a significant difference in the practical application of such filters, where the cost of inaccuracies can be high. Performance Comparison of—Filters With and Without ANN, Including Percentage Increase as shown in Fig 12.

### 4.5 Comparative analysis of NN-Kalman and NN-Alpha-Beta filters

The comparative analysis of the Neural Network-based Kalman filter (NN-Kalman) and the Neural Network-based Alpha-Beta filter (NN-Alpha-Beta) begins with their initial performance metrics, where both demonstrated high Root Mean Square Error (RMSE) values—5.216 for the NN-Kalman and 5.21 for the NN-Alpha-Beta. This similarity in initial RMSE indicated a comparable level of inaccuracy due to noisy data inputs.

With the integration of neural network-based learning algorithms, marked improvements were observed in both filters. The NN-Kalman filter’s RMSE was significantly reduced by 54.22% upon optimizing the *R* (noise covariance) and *F* (error factor) parameters, demonstrating its advanced ability to refine predictions and manage complex datasets. This optimization reflects the system’s sophisticated learning capability, which involves fine-tuning to align closely with diverse and complex data patterns, making it well-suited for complex applications requiring high accuracy and detailed data processing.

Meanwhile, the NN-Alpha-Beta filter achieved a 38.2% reduction in RMSE, a notable improvement facilitated by the dynamic adjustment of the alpha and beta parameters. This indicates a significant boost in predictive capability, afforded by a simpler learning algorithm that iteratively adapts these parameters. The simpler optimization process, while yielding a smaller improvement than the NN-Kalman, emphasizes the filter’s robustness and user-friendly nature, catering to scenarios where computational efficiency and speed are prioritized over ultimate precision. Table 4 shows a clear representation of improvements in filter performance.

**Table 4:** Comparative analysis of initial sensor readings with NN-Kalman filter and alpha beta filter.

Performance Metric	Sensor Readings	NN-Kalman Filter	NN-Alpha Beta Filter
RMSE	5.21	2.38	3.22

Incorporating a neural network with a Kalman filter, wherein the sensor noise covariance (R) is dynamically adjusted while maintaining a consistent process error covariance (F), can enhance the filter’s performance and precision. This fusion enables the system to adapt more responsively to variations in sensor readings, achieving superior accuracy under certain conditions. Conversely, the alpha-beta filter operates through the careful tuning of its two parameters, alpha and beta, which govern the system’s responsiveness to changes in measurements and trends, respectively. This approach allows for a more agile response to dynamic situations, making the system robust in environments where direct control over responsiveness to measurement noise and maneuvering is essential.

**Fig 13:**
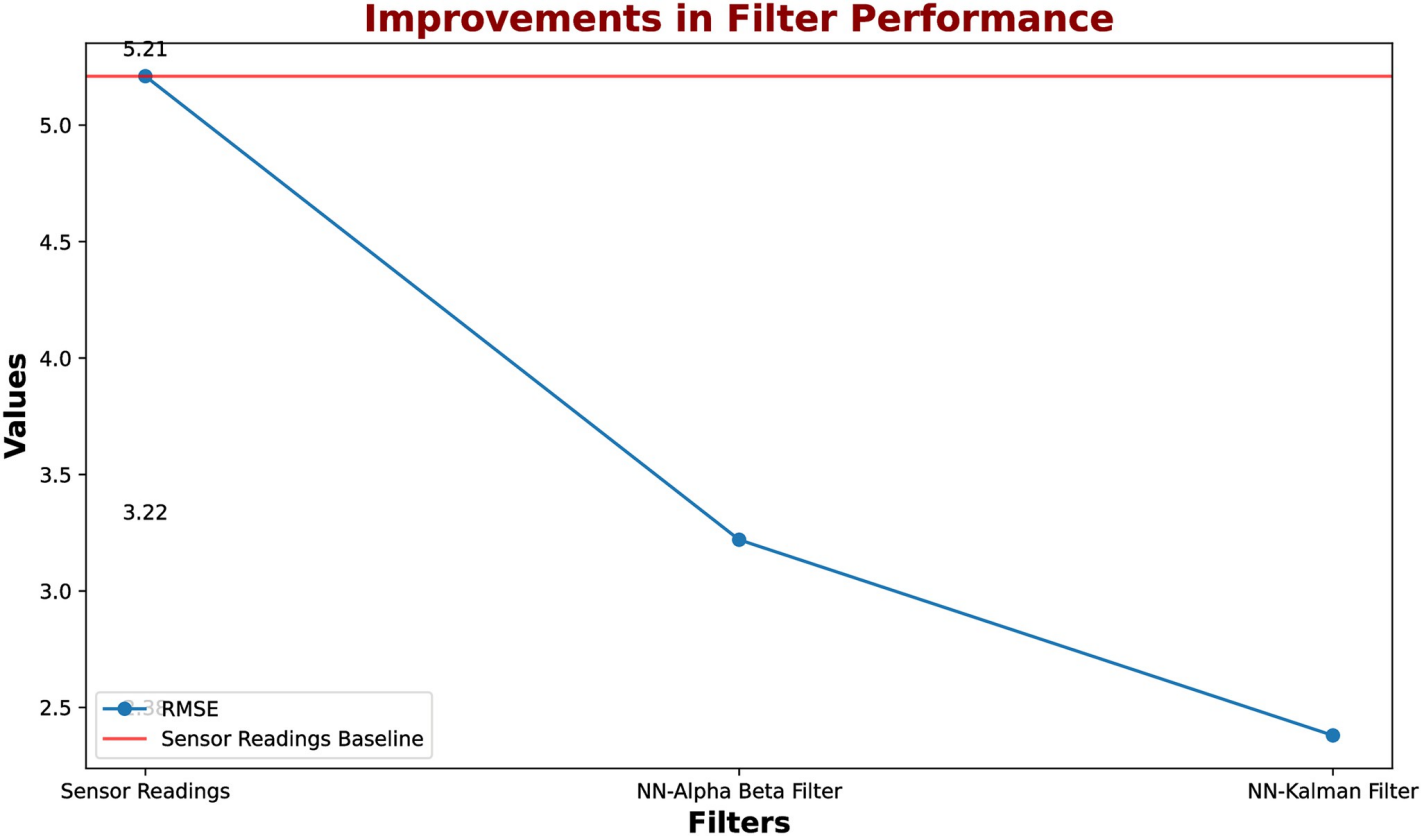
Representation of very high initial error value, and reduced error based on the NN-Kalman filter and alpha beta filter.

In terms of application, the choice between the NN-Kalman and NN-Alpha-Beta filters is determined by specific needs. The NN-Kalman filter is better suited for applications demanding maximum prediction accuracy and the processing of complex, multi-variable data. Conversely, the NN-Alpha-Beta filter, with its more accessible parameter adjustments and lower computational demands, may be more appropriate for applications where a balance of simplicity, speed, and predictive accuracy is needed. Fig 13 shows the very high initial error value, and reduced error based on the NN-Kalman filter and alpha beta filter.Table 5 shows the improvements in filter performance.

**Table 5:** Improvements in filter performance.

Performance Metric	Improvement in Kalman Filter	Improvement in Alpha Beta Filter
**RMSE**	**53.41%**	**38.2%**

### 4.6 Comparison with other existing methods

Table 6 presents a comparative analysis of the Root Mean Square Error (RMSE) across different filtering methods. The conventional method exhibits the highest RMSE at 5.216, indicating less accuracy in performance. In contrast, the *α*-*β* Filter with Deep Belief Network (DBN), Deep Extreme Learning Machine (DELM), and Support Vector Machine (SVM) show improved results, with RMSE values of 3.605, 3.901, and 4.015, respectively, as presented in [9]. The proposed methods, including the NN-Kalman Filter and NN-Alpha Beta Filter, show superior accuracy, with the lowest RMSE values of 2.38 and 3.22, respectively. This indicates that the proposed neural network-based approaches significantly enhance the filtering accuracy compared to conventional and other existing methods.

**Table 6:** Comparison of proposed method with other existing methods.

Performance Measure	RMSE
Conventional Method	5.216
*α*-*β* Filter with DBN [9]	3.605
*α*-*β* Filter with DELM [9]	3.901
*α*-*β* Filter with SVM [9]	4.015
Proposed NN-Kalman Filter	2.38
Proposed NN-Alpha Beta Filter	3.22

The performance improvement observed in the proposed methods can be attributed to several factors, especially when comparing them with models like the *α*-*β* Filter integrated with DBN or SVM. One key reason for the superior performance of the proposed NN-Kalman and NN-Alpha Beta Filters is the deeper integration between the neural network and filtering techniques. In the case of the *α*-*β* Filter with DBN and SVM, the integration relies on traditional training models, which may not fully exploit the benefits of adaptive learning during the filtering process. These methods tend to treat filtering and machine learning components as somewhat independent processes, where the filter refines inputs for the machine learning model, but the two are not tightly coupled. On the other hand, the proposed methods employ a more sophisticated coupling of the neural network with the filtering techniques. Specifically, the integration method used in the proposed NN-Kalman and NN-Alpha Beta Filters is more deeply synergized, wherein the neural network learns not only from the input data but also optimizes the filtering process itself. This bidirectional learning ensures that both the filtering mechanism and the network evolve concurrently, leading to more accurate predictions and reduced RMSE values.

Also, the different network architectures contribute to this performance boost. The neural network structure used in the proposed methods is specifically designed to work with filtering systems like Kalman and *α*-*β*. The architecture is optimized to capture and adapt to the dynamic nature of the system’s uncertainties, a feature that traditional methods such as DBN and SVM lack. DBN and SVM are more generalized machine learning models, which, while powerful, are not specifically tailored for the filtering tasks in question. In contrast, the proposed methods involve custom neural architectures that can better represent the temporal and noise patterns present in the data, thereby significantly enhancing filtering accuracy. Furthermore, the proposed methods utilize adaptive learning techniques that are inherently more flexible than the static models used by DBN and SVM. These static models are trained on a fixed dataset and lack the capacity to adjust dynamically to new or changing data in real-time. In contrast, the proposed neural network approaches are capable of continuous learning, enabling them to better handle non-stationary and noisy data, which results in a notable reduction in RMSE.

**Fig 14:**
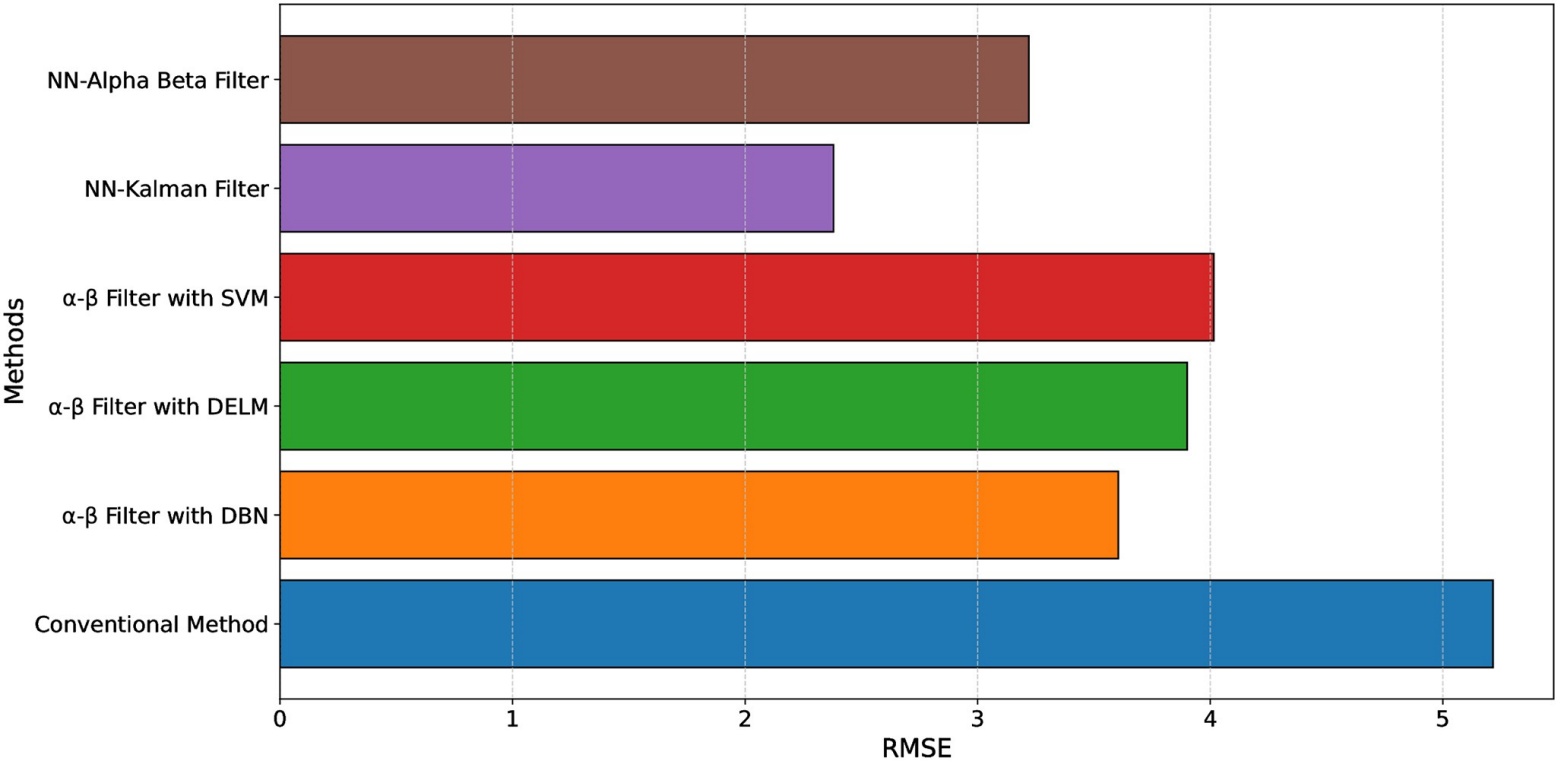
Comparison of proposed method with other existing methods.

The difference in the network structures is also important. While the DBN and SVM models rely on standard feedforward architectures, the proposed NN-Kalman and NN-Alpha Beta Filters incorporate recurrent neural networks (RNNs) or other advanced architectures that are more adept at handling sequential data. This ability to process data sequences more effectively enhances the prediction and filtering accuracy of the system. The combination of these advanced network architectures with optimized filtering strategies explains the significant reduction in RMSE compared to existing methods. Therefore, the performance improvement of the proposed methods is not merely due to the application of neural networks but also to the careful design of the integration between the neural networks and the filtering algorithms. This tailored integration ensures that the proposed methods can learn and adapt more effectively than the traditional models, resulting in superior accuracy. Fig 14 shows a comparative analysis of the conventional, existing and proposed methods.

### 4.7 Discussion

The integration of neural networks into traditional filtering algorithms such as the Kalman filter and the alpha-beta filter represents a significant advancement in dynamic system analysis. This study has demonstrated that by dynamically tuning filter parameters using neural networks, both the adaptability and accuracy of these systems can be substantially improved under varying conditions.

The neural network-based alpha-beta filter showed a 38.2% improvement in prediction accuracy, while the neural network-enhanced Kalman filter achieved a 53.4% increase. These improvements underscore the effectiveness of our approach in dynamically responding to changes in system dynamics, significantly surpassing the performance of traditional filtering methods. Such enhancements are critical in applications where system conditions are continually changing and where the accuracy of predictions is utmost.

However, the introduction of neural networks also brings additional complexity and potential drawbacks. One notable concern is the risk of overfitting, particularly when neural networks are trained on highly specific or non-representative datasets. This risk was diminished to some extent by the careful preprocessing and normalization of data, yet the possibility remains, especially in systems with complex or irregular patterns. Moreover, the computational demands of neural networks may limit their applicability in resource-constrained environments, suggesting a need for optimized neural network architectures that maintain performance while reducing computational overhead.

Another critical aspect is the impact of data biases. The performance of the neural network-driven filters depends heavily on the representativeness of the training data. Biases in the data can lead to suboptimal parameter tuning and, consequently, less accurate predictions. Future work should focus on enhancing the robustness of these models by expanding the datasets to include a wider array of conditions and by implementing adaptive learning techniques that can update the training data based on new inputs continuously.

The comparative analysis between the enhanced and traditional filters highlighted not only the improved performance but also the conditions under which each model surpasses. This information is vital for users to understand the operational contexts in which these enhanced models would provide the most benefit. Lastly, the significant improvements predictable in this paper indicate a promising direction for future research. Further development could explore the integration of other types of neural network architectures, such as recurrent neural networks, which might offer advantages in handling time-series data typical of dynamic systems. Also, real-world applications of these models could provide valuable feedback that would fuel continuous improvements and refinements.

## 5 Conclusions

This paper introduces an innovative prediction model based on neural networks, designed to improve the performance of the Kalman filter and the alpha-beta filter algorithms under dynamic conditions. The models enable these traditional filters to dynamically adjust their parameters by continuously monitoring their effectiveness. We have developed and assessed a neural network-based Kalman filter and a neural network-based alpha-beta filter, each incorporating a neural network to enhance prediction accuracy. The alpha-beta filter algorithm now includes two output parameters, *α* and *β*, while the Kalman filter algorithm uses an internal parameter, *R*, to estimate error and adjust the noise factor, *F*. Both the noise factor and internal parameters are modifiable within this model. The effectiveness of these advanced filters was gauged using the root mean square error (RMSE) metric.

Initially, both algorithms reported a high RMSE of 5.21. Post enhancement, the neural network-based alpha-beta filter reduced the RMSE to 3.22, indicating a 38.2% improvement in accuracy. The neural network-based Kalman filter exhibited even better performance, with an RMSE of 2.38 when *R* was set to 20 and *F* to 0.02, reflecting a 53.4% increase in accuracy. This demonstrates that the proposed neural network-enhanced systems substantially surpass the accuracy of the simple Kalman and alpha-beta filters.

Moreover, our comparative analysis reveals that while the Kalman filter initially shows higher accuracy than the alpha-beta filter, its performance varies and is sensitive to changes in *R* and *F*. However, when evaluating the overall performance across various tests, the alpha-beta filter, when augmented with the neural network model, demonstrates superior performance.
